# The effect of injectable platelet-rich fibrin and platelet-rich fibrin in regenerative endodontics: a comparative *in vitro* study

**DOI:** 10.1590/1678-7757-2023-0449

**Published:** 2024-06-14

**Authors:** PAN Jing, LUO Linjuan, Zhen JIANG, Haiyan HUANG, Beizhan JIANG

**Affiliations:** 1 Shanghai Engineering Research Center of Tooth Restoration and Regeneration Stomatological Hospital and Dental School Tongji University Shanghai China Shanghai Engineering Research Center of Tooth Restoration and Regeneration , Stomatological Hospital and Dental School of Tongji University , Department of Pediatric Dentistry, Shanghai , China .

**Keywords:** Platelet-rich fibrin, Angiogenesis, Mineralization, Regenerative endodontics

## Abstract

**Objective:**

To explore the feasibility of injectable platelet-rich fibrin (i-PRF) in regenerative endodontics by comparing the effect of i-PRF and platelet-rich fibrin (PRF) on the biological behavior and angiogenesis of human stem cells from the apical papilla (SCAPs).

**Methodology:**

i-PRF and PRF were obtained from venous blood by two different centrifugation methods, followed by hematoxylin-eosin (HE) staining and scanning electron microscopy (SEM). Enzyme-linked immunosorbent assay (ELISA) was conducted to quantify the growth factors. SCAPs were cultured with different concentrations of i-PRF extract (i-PRFe) and PRF extract (PRFe), and the optimal concentrations were selected using the Cell Counting Kit-8 (CCK-8) assay. The cell proliferation and migration potentials of SCAPs were then observed using the CCK-8 and Transwell assays. Mineralization ability was detected by alizarin red staining (ARS), and angiogenesis ability was detected by tube formation assay. Real-time quantitative polymerase chain reaction (RT-qPCR) was performed to evaluate the expression of genes related to mineralization and angiogenesis. The data were subjected to statistical analysis.

**Results:**

i-PRF and PRF showed a similar three-dimensional fibrin structure, while i-PRF released a higher concentration of growth factors than PRF ( *P* <.05). 1/4× i-PRFe and 1/4× PRFe were selected as the optimal concentrations. The cell proliferation rate of the i-PRFe group was higher than that of the PRFe group ( *P* <.05), while no statistical difference was observed between them in terms of cell mitigation ( *P* >.05). More importantly, our results showed that i-PRFe had a stronger effect on SCAPs than PRFe in facilitating mineralization and angiogenesis, with the consistent result of RT-qPCR ( *P* <.05).

**Conclusion:**

This study revealed that i-PRF released a higher concentration of growth factors and was superior to PRF in promoting proliferation, mineralization and angiogenesis of SCAPs, which indicates that i-PRF could be a promising biological scaffold for application in pulp regeneration.

## Introduction

Immature permanent teeth with pulp necrosis always lose physiological functions, in which case incomplete root development may cause root fracture, and open apical foramen is more susceptible to infection from periapical and periodontal tissues. ^1^ Given the adverse effects of traditional endodontic treatments for pulp necrosis, advances in regenerative medicine have paved the way for biologically regenerative endodontic procedures. ^2^ Clinically, cell homing is the main method used in regenerative endodontics, in which a specialized microenvironment is created at the root canal injury to allow for host cell recruitment and activity. ^3^

Currently, one of the major challenges of this strategy is to find a beneficial material combined with scaffold and growth factors, and autologous blood products have attracted considerable interest in this regard. Platelet-rich fibrin (PRF), the second generation platelet concentrate, contains a supraphysiological concentration of platelets compared with whole blood and is a reservoir of many growth factors for regeneration. ^4^ Recent cell experiments concluded that PRF can improve cell proliferation, migration or differentiation of stem cells from the apical papilla (SCAPs), gingival fibroblasts and periodontal ligament fibroblasts. ^5 - 8^ Some clinical studies also found that PRF brought great improvement in root development and restoration of tooth vitality. ^9 - 13^

Although PRF has complete immune biocompatibility, with various *in vitro* and *in vivo* studies revealing its effect on pulp regeneration, ^10 , 12 , 14^ its application requires relatively large space, which limits its use to narrow teeth root canals, unlike an injectable form. ^15^ Interestingly, the low-speed centrifugation concept (LSCC) introduced a novel derivative of PRF: injectable platelet-rich fibrin (i-PRF). ^16^ The high penetration ability of i-PRF suggests that it may be an ideal material for pulp regeneration, as i-PRF could be easily injected into root canals and completely fill the voids. Previous reports indicated that i-PRF shows a three-dimensional fibrin scaffold with platelets, leukocytes, type I collagen, osteocalcin and growth factors, ^17^ which can mimic the extracellular matrix to some extent and favor cell adhesion and biological functions. ^18^ Other studies also demonstrated the ability of i-PRF to regulate inflammatory response, ^17^ resist bacterial activity ^19^ and promote osteogenesis. ^20 , 21^ Such properties suggest that the use of i-PRF in the regeneration process of soft and mineralized tissues is promising. The clinical operability of i-PRF is superior to that of PRF, but its role in regenerative endodontics has not been fully investigated. Therefore, in this *in vitro* study, we compared the structural composition, growth factor content, and effects of i-PRF and PRF on cell proliferation, migration, mineralization and angiogenesis of SCAPs to explore the feasibility of i-PRF in pulp regeneration.

## Methodology

This study was carried out under the guidance of the Institutional Review Board of the Stomatological Hospital and Dental School of Tongji University, China, and was approved by the Ethics Committee ([2021]-DW-70). Written informed consent was obtained from each volunteer.

### Histological and morphological analysis of i-PRF and PRF

Throughout this study, ten healthy volunteers (male, 18-35 years old, non-smokers, non-alcohol consumers, without systemic diseases and without recent use of medications affecting the blood system) were recruited for venous blood extraction. After obtaining informed consent,10 mL of venous blood was collected from each volunteer in a sterile vacuum glass tube without anticoagulants. In accordance with previously reported protocols, ^4 , 22^ the tube was immediately centrifuged at 700 rpm for 3 min at 4°C to obtain upper i-PRF or at 3000 rpm for 10 min to obtain upper PRF using a centrifuge (Thermo, USA). Three of ten venous blood samples were randomly selected and divided into three parts for histological study, morphological study and growth factor determination, respectively. For histological study, the gelled i-PRF or PRF membrane was fixed in 4% paraformaldehyde, embedded in paraffin and sectioned at 4 μm thickness for hematoxylin-eosin (HE) staining (Keygen, Nanjing, China). For morphological study, the samples were fixed with 2.5% glutaraldehyde for 1 h and then dehydrated in a vacuum freeze dryer (Thermo Scientific, Germany) overnight. A scanning electron microscope (SEM, S-4800, Hitachi, Tokyo, Japan) was used to capture the representative images.

### Quantification of growth factors in i-PRF and PRF

Growth factors were harvested as described previously with slight modifications. ^5 , 23^ The isolated i-PRF or PRF membrane was briefly frozen overnight in a vacuum freeze dryer. The lyophilized i-PRF or PRF was pulverized and soaked in 20 mL α-modified Eagle’s medium (α-MEM, Hyclone, Logan, UT) at 4°C for 24 h. After centrifugation and filtration, an enzyme-linked immunosorbent assay (ELISA, R & D, Minneapolis, MN, USA) was performed to quantify four growth factors in the extracted solution, including platelet-derived growth factor-BB (PDGF-BB), insulin-like growth factor-1 (IGF1), transforming growth factor- β1 (TGF- β1) and vascular endothelial growth factor (VEGF).

Isolation and characterization of SCAPs

The healthy apical papillae were isolated from the healthy third molars without apical closure, which were extracted from five volunteers (14-25 years old) at the Department of Oral and Maxillofacial Surgery of the Stomatological Hospital and Dental School of Tongji University after obtaining informed consent. The apical papillae were immediately minced into small pieces and digested with 3 mg/mL collagenase type I (Sigma-Aldrich, St Louis, MO, USA) under sterile conditions for cell harvest. The tissues and cells were cultured in α-MEM supplemented with 10% fetal bovine serum (FBS, Gibco BRL, Gaithersburg, MD) and 1% antibiotic (100 U/mL penicillin-100 mg/mL streptomycin, Sigma-Aldrich) at 37 °C in an atmosphere of 5% CO _2_ and 95% humidity. Third to fourth passages of SCAPs were used for cell experiments. Flow cytometric analysis was conducted to characterize the immunophenotype of SCAPs as previously described ^24^ , assessing the expression of mesenchymal stem cell markers (CD45, CD 90, and CD146).

### Optimal concentrations of i-PRF extract (i-PRFe) and PRF extract (PRFe)

In accordance with the aforementioned experimental protocol, ^25^ the pulverized i-PRF or PRF was soaked in 50 mL α-MEM at 4 °C for 24 h. After centrifugation, the medium was filtered to obtain 1× i-PRFe or 1× PRFe. Fresh medium was prepared every 2 days. Four concentrations of i-PRFe and PRFe (1×, 1/2×, 1/4× and 1/8×) supplemented with 10% FBS and 1% antibiotic were used to select the optimal concentrations by Cell Counting Kit-8 (CCK-8, Keygen, Nanjing, China). The SCAPs (2×10 ^3^ cells/well) were briefly seeded in 96-well plates (Corning, NY, USA) for 12 h with three multiple holes in each group, then treated with different concentrations of i-PRFe or PRFe for 1, 3 and 5 days, respectively, followed by the addition of 10 μL CCK-8 reagent per 100 μL culture medium. Medium without any extract was used as the control group. Cell counts were analyzed using a microplate reader (Bio-Tek, Hercules, CA) at a wavelength of 450 nm. After extensive analysis of the results, the optimal concentrations of i-PRFe and PRFe were selected for the cell experiments described below.

Cell proliferation assay and cell migration assay

Cell proliferation was measured using the CCK-8 assay as described above, and samples were divided into 3 groups: control group; optimal concentration of i-PRFe group; and optimal concentration of PRFe group. Cell migration was observed using Transwell filter inserts (Corning). A suspension of SCAPs (1×10 ^5^ cells/mL) was added to the upper chamber of the filter, and i-PRFe or PRFe was added to the lower chamber. α-MEM with or without 10% serum was prepared as positive and negative control groups. Each group was provided with three multiple holes. After 24 h, cells that migrated to the lower chamber were fixed and stained with crystal violet (Sigma-Aldrich). Cells remaining in the upper chamber were gently abraded with a cotton swab. Representative images were captured randomly in three microscope fields using a light microscope. The staining was then removed with 3% acetic acid solution, and the OD values were determined using a microplate reader at a wavelength of 570 nm.

### Mineralization induction and Alizarin Red Staining (ARS)

ARS was performed to detect the formation of mineralization nodules after mineralization induction. The control odonto/osteogenic medium (OM) contained 0.05 mM ascorbic acid, 100 nM dexamethasone, 10 mM β-glycerophosphate (Sigma-Aldrich), 10% FBS and 1% antibiotic in α-MEM as previously described, with slight modifications. ^26^ Samples were divided into four groups with three multiple holes in each group: control group; OM group; and i-PRFe or PRFe with OM groups (experimental groups). Cells (1×10 ^5^ cells/well) were seeded in 6-well plates (Corning) and cultured with different media for 7, 14 and 21 days. For ARS, paraformaldehyde-fixed cells were stained with 2% ARS solution (Sigma-Aldrich) and photographed under a microscope after several washes. Staining was then removed with 3% acetic acid solution and the OD values were determined using a microplate reader at a wavelength of 570 nm.

### Angiogenesis assay and Immunofluorescent (IF) staining

A tube formation assay was performed to detect the formation of tubular structures after angiogenesis induction according to previously reported protocols, with slight modifications. ^27^ Angiogenic medium (AM) containing 50 µg/L VEGF (Pepro Tech, USA), 10 µg/L basic fibroblast growth factor (b-FGF, Pepro Tech) and 1% antibiotic in α-MEM was used as a positive control. The samples were divided into four groups with three multiple holes in each group: control group; AM group; i-PRFe group and PRFe group (experimental groups). SCAPs were seeded in 6-well plates (Corning) and cultured with different media for 7 days before being suspended in FBS-free conditioned media at 2 × 10 ^5^ cells/mL. Matrigel Basement Membrane Matrix (10 µL per well) (Corning Inc, Corning, NY, USA) was added to the lower chamber of Slide Angiogenesis (ibidi, Italy) and allowed to polymerize at 37 °C for at least 30 min. Cell suspension (50 µL per well) was inoculated on the surface of each solidified Matrigel and cultured at 37 °C in an atmosphere of 5% CO _2_ for 6 h. For IF staining, the fixed cells were stained with calcein (Invitrogen, Carlsbad, CA, USA) to make the network more visible and photographed under a Nikon camera attached to a fluorescence microscope. For the fluorescence images, ImageJ software (version 10.2; National Institutes of Health, Bethesda, MD, USA) was used to quantify the extent of tubular formation in each replicate well.

Real-time quantitative polymerase chain reaction (RT-qPCR)

**Figure 1:**
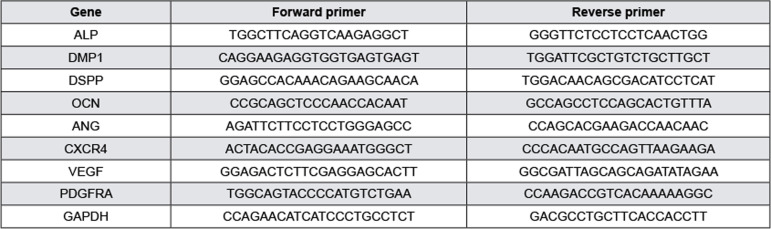
Primer sequences used in the RT-qPCR reaction

RT-qPCR was performed to evaluate the expression of genes related to mineralization and angiogenesis. To isolate total RNA, cells were briefly lysed with Trizol reagent (TaKaRa, Kusatsu, Japan) according to the manufacturer’s instructions. The extracted RNA was reverse transcribed using a complementary DNA synthesis kit (TaKaRa), and the relative messenger RNA expression of the target gene was analyzed using HieffTM qPCR ^®^ SYBR Green Master Mix (Applied Biosystems, Foster City, CA, USA). *Glyceraldehyde-3-phosphate dehydrogenase* ( *GAPDH* ) was used as a control to normalize RNA expression levels. Figure 1 shows the primer sequences for *alkaline phosphatase* ( *ALP* ), *dentin matrix protein 1* ( *DMP1* ), *dentin sialophosphoprotein* ( *DSPP* ), *osteocalcin* ( *OCN* ), *angiogenin* ( *ANG* ), *C-X-C motif chemokine receptor 4* ( *CXCR4* ), *VEGF* and *platelet-derived growth factor receptor alpha* ( *PDGFRA* ) (Sango Biotech, Shanghai, China).

### Statistical analysis

All experiments were performed in triplicate. Statistical analysis was performed using SPSS version 20.0 (IBM SPSS, Armonk, NY, USA). The graphics software used was GraphPad Prism 6.0 (GraphPad Software, La Jolla, CA, USA). Data were presented as mean ± standard deviation (SD) and subjected to one-way analysis of variance (ANOVA). *P* <.05 was considered statistically significant.

## Results

### Histological and morphological analysis of i-PRF and PRF

**Figure 2:**
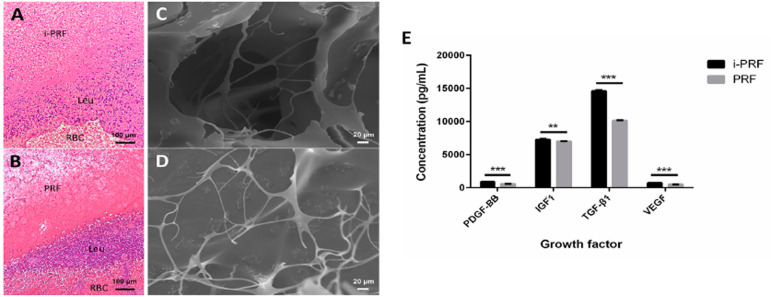
Structures and growth factors of i-PRF and PRF. The microstructures of i-PRF (A) and PRF (B) were observed by hematoxylin-eosin (HE) staining, in which fibrin fibrils were stained pink, while platelets and leukocytes were stained dark blue (scale bar = 100 µm). Leu: leukocytes; RBC: red blood cells. The ultrastructures of i-PRF (C) and PRF (D) were observed by scanning electron microscopy (SEM) (scale bar = 20 µm). (E) The growth factors PDGF-BB, IGF1, TGF-β1 and VEGF were detected by enzyme-linked immunosorbent assay (ELISA). Results are expressed as mean ± standard deviation. (**P<.01, ***P<.001)

This part of the experiment was a descriptive analysis of i-PRF and PRF. After centrifugation, the blood sample was roughly divided into two layers, and liquid i-PRF or PRF clot was located to the upper layer. HE staining revealed that i-PRF and PRF had a similar structure, with a platelet-rich fibrin network in the upper layer, residual red blood cells in the lower layer, and a leukocyte accumulation zone at the interface between the two layers (Figure 2A and B). The density of leukocytes and platelets gradually increased from the i-PRF or PRF zone to the interface zone. And regardless of the interface zone, the cellular components in the upper i-PRF seemed to be denser than those in the upper PRF. In terms of ultrastructure, SEM images of both i-PRF and PRF showed a loose and porous three-dimensional organization of the fibrin network (Figure 2C and D).

### Quantification of growth factors in i-PRF and PRF

The content of growth factors in i-PRF and PRF was quantified using ELISA (Figure 2E). PDGF-BB, IGF1, TGF-β1 and VEGF were all detected in i-PRF and PRF, while the concentrations of four growth factors were significantly higher in i-PRF than in PRF ( *P* <.05).

### Optimal concentrations of i-PRFe and PRFe

**Figure 3:**
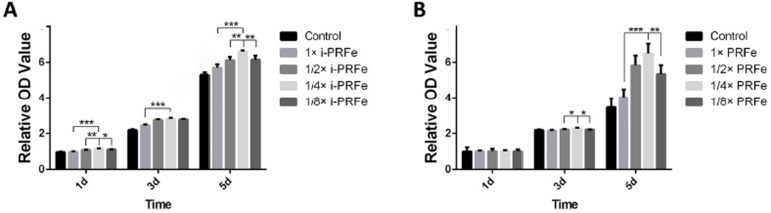
Effect of different concentrations of i-PRFe (A) and PRFe (B) on the proliferation of SCAPs for 1, 3 and 5 days. Results are expressed as mean ± standard deviation of the OD value. (*P<.05, **P<.01, ***P<.001)

After being cultured at four different concentrations for 1, 3 and 5 days, i-PRFe promoted better proliferation values than those of the control group, and among the five groups, the 1/4× i-PRFe group had the best effect on the proliferation of SCAPs ( *P* <.05, Figure 3A). As for PRFe, the cell proliferation value of the 1/4× PRFe group was higher than that of the 1/2× PRFe and 1/8× PRFe groups on day 3, and a significant improvement was also observed in the 1/4× PRFe group compared with the 1× PRFe and 1/8× PRFe groups on day 5 ( *P* <.05, Figure 3B). Therefore, 1/4× i-PRFe and 1/4× PRFe were selected as the optimal concentrations and used in the cell experiments described below.

Effect of i-PRFe and PRFe on the proliferation and migration of SCAPs

**Figure 4:**
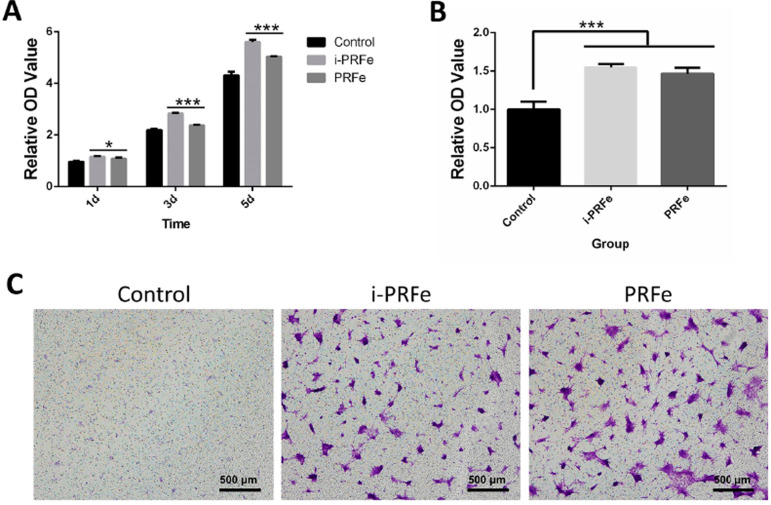
Evaluation of proliferation and migration of SCAPs after culturing with i-PRFe or PRFe. (A) Statistical analysis of the proliferation proportions of different groups after culturing for 1, 3 and 5 days. Results are expressed as mean ± standard deviation of the OD value. (B) The average number of migrating cells from different groups per field was statistically analyzed after 24 h. Results are expressed as mean ± standard deviation of the OD value. (C) The representative images of the crystal violet-stained migratory cells from different groups were captured by light microscopy (scale bar = 500 µm). (*P<.05, ***P<.001)

In the cell proliferation assay, SCAPs in the experimental groups showed higher proliferation rates than those in the control group after being cultured for 1, 3 and 5 days, and a significant improvement was also observed in the i-PRFe group compared with the PRFe group ( *P* <.05, Figure 4A). The result of the Transwell assay showed that the migratory cells in both i-PRFe and PRFe groups were denser than those in the control group after being cultured for 24 h ( *P* <.05, Figure 4C), but no statistical difference was observed between the experimental groups ( *P* >.05, Figure 4B).

Effect of i-PRFe and PRFe on odonto/osteogenic differentiation of SCAPs

**Figure 5:**
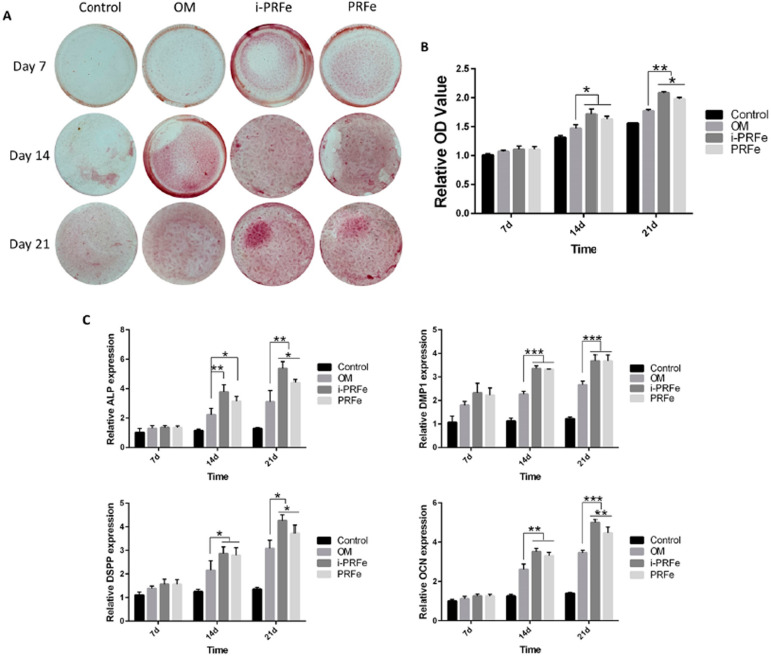
Effect of i-PRFe and PRFe on odonto/osteogenic differentiation of SCAPs. (A) Representative images of alizarin red staining (ARS) of SCAPs after culturing with different media for 7, 14 and 21 days. (B) Mineralization quantification was analyzed, and the results are expressed as mean ± standard deviation of the OD value. (C) Relative gene expression levels of ALP, DMP-1, DSPP and OCN after culturing with different media for 7, 14 and 21 days. Results are expressed as mean ± standard deviation. (*P<.05, **P<.01, ***P<.001)

After incubation with different media, ARS showed that the positive staining became denser after 14 and 21 days of the co-treatment of i-PRFe or PRFe with OM, and the staining in the i-PRFe with OM group was stronger compared with the PRFe with OM group on day 21 ( *P* <.05, Figure 5A and B). Consistent with this, the RT-qPCR results showed that i-PRFe or PRFe with OM could noticeably upregulate the mRNA expression levels of four odontogenic genes compared with the OM group after induction for 14 and 21 days, and the relative expression of *ALP* , *DSPP* and *OCN* in the i-PRFe with OM group was much higher than in the PRFe with OM group on day 21 ( *P* <.05, Figure 5C).

Effect of i-PRFe and PRFe on angiogenic differentiation of SCAPs

**Figure 6:**
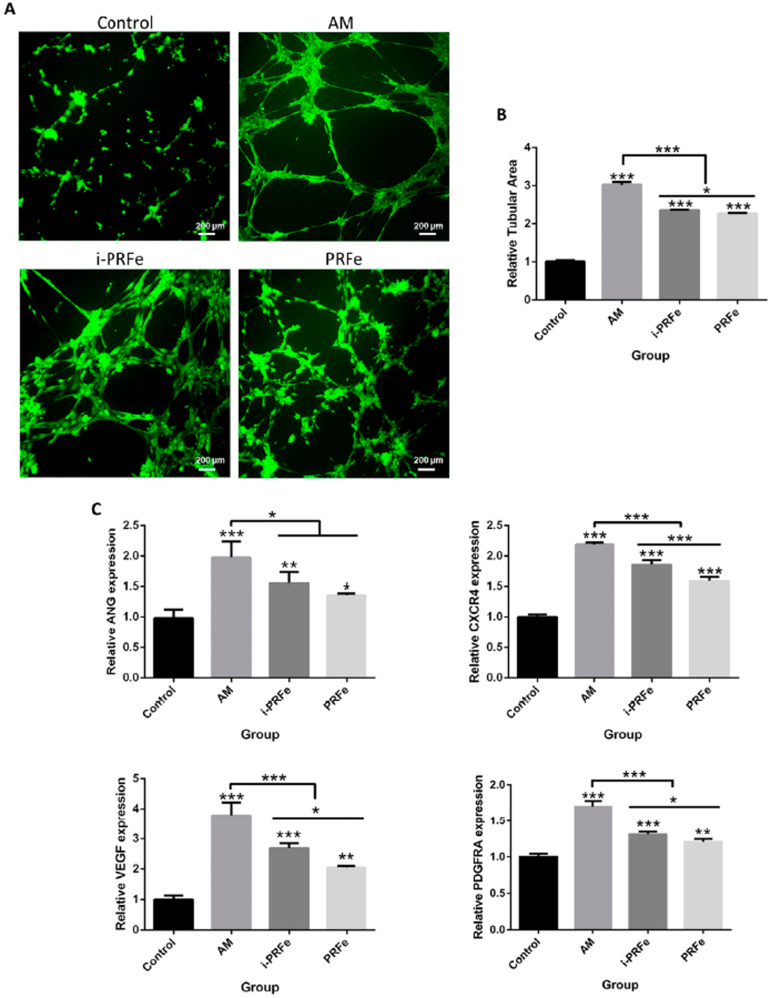
Effect of i-PRFe and PRFe on angiogenic differentiation of SCAPs. (A) Representative images of immunofluorescence (IF) staining of SCAPs after culturing with different media for 6 h. (B) The quantification of angiogenesis was determined using ImageJ software, and the results were shown with the ratio of tubular area to total area. (C) Relative gene expression levels of ANG, CXCR4, VEGF and PDFGFRA after culturing with different media for 6 h. Results are expressed as mean ± standard deviation. (*P<.05, **P<.01, ***P<.001)

A tube formation assay was conducted to determine the effect of i-PRFe and PRFe on angiogenesis. Compared with the control group, IF staining revealed that the tubular areas became larger after treatment with i-PRFe or PRFe, and more tubes were formed in the i-PRFe group than in the PRFe group ( *P* <.05, Figures 6A and B). Similarly, the mRNA expression levels of *ANG* , *CXCR4* , *VEGF* and *PDGFRA* were upregulated by i-PRFe or PRFe. Except for *ANG* , the gene expression levels were higher in the i-PRFe group than in the PRFe group ( *P* <.05, Figure 6C). However, the effect of the experimental groups on tubular formation and angiogenesis-related gene expression was not as superior as that of the AM group (positive control) ( *P* <.05, Figure 6A, B and C).

## Discussion

Dental pulp regeneration via cell homing requires a biocompatible scaffold where recruited stem cells can be induced to proliferate, migrate and differentiate under the regulation of growth factors. ^28^ Clinical studies have shown that PRF scaffold achieved favorable outcomes in guided endodontic repair. ^29^ As a derivative of PRF, i-PRF also has the potential to promote regeneration with the additional advantage of liquidity, but few studies on its application in pulp regeneration were found. ^30^ Therefore, this study compared the biological behaviors of SCAPs when treated with i-PRFe or PRFe to explore the possibility of novel i-PRF in dental pulp regeneration, especially angiogenesis to restore blood supply, which is a critical event during the regenerative phase.

The characterization of scaffold material is an important procedure to understand cellular and clinical effects. ^31^ In this study, histological staining revealed that i-PRF and PRF had a similar structure and composition, and the platelets and leukocytes in the upper i-PRF layer seemed to be denser than those in the upper PRF layer. Choukroun, et al. ^32^ (2018) observed that the mass, size and density range of leukocytes and platelets required a low relative centrifugal force (RCF), which was sufficient to separate them from the remaining blood components while retaining them in the upper layer. Due to the shorter centrifugation time and lower centrifugal force, more leukocytes and platelets with lower gravity were left in the upper i-PRF than in PRF, and the HE staining results confirmed this. Higher concentrations of platelets and leukocytes contribute to the regenerative process because platelets play a crucial role in the early stage of wound healing by exerting their synergistic effect with the fibrin network, and leukocytes attract various types of cells to the defect areas. ^33^ As for the result of SEM, representative images showed that the three-dimensional fibrin network in i-PRF and PRF was loose and porous, which may facilitate cell penetration, allowing various types of cells to migrate into the scaffold and generate soft and mineralized tissue. ^34^

Growth factors are essential signaling molecules that mobilize endogenous cells and direct cellular proliferation and differentiation during the regenerative process. In pulp regeneration, these molecules can be secreted by cells in the periapical tissues, mobilized from adjacent dentin, or delivered by platelet concentrates such as i-PRF and PRF. ^35^ In this study, i-PRF was found to release a higher concentration of PDGF-BB, IGF1, TGF-β1 and VEGF than PRF. This finding was consistent with J Choukroun, et al. ^32^ (2018), whose data showed that LSCC enriched the growth factors VEGF and TGF-β1 in fluid PRF-based matrices. The increased concentration of growth factors was likely related to the increased number of platelets and leukocytes, as these cells are important sources of growth factors. Some recent studies have focused on the introduction of exogenous growth factors in cell experiments or animal models, but there are still unanswered questions regarding the selection and concentration of signaling molecules the expensive price and the instability of bio-conductive molecules. ^35^ Therefore, we believe that taking advantage of regenerative factors in autologous blood products for regenerative engineering is the feasible method at present.

In the cell homing strategy, SCAPs have been shown to be the most likely endogenous stem cells to migrate into the root canal ^36^ and were therefore chosen in this study. Considering that possible cytotoxicity, higher growth factor content and more platelets do not necessarily mean a stronger positive biological effect on tissue regeneration, the indication of a dose-dependent effect of platelet concentrates is rarely emphasized in the literature. ^37^ In addition, our previous study found that 1/4× i-PRFe had the best effect on the proliferation of SCAPs, and 1/4× i-PRFe could also significantly improve the migration and mineralization abilities of SCAPs. ^25^ Therefore, four concentration gradients of 1×, 1/2×, 1/4× and 1/8× were set in this study to select the optimal concentrations for promoting cell proliferation. The result of the present study also showed that 1/4× i-PRFe and 1/4× PRFe had the best effect on the proliferation of SCAPs. Therefore, 1/4× was chosen to conduct the final experiments. When comparing the effect on cell biological behaviors, the results showed that both i-PRFe and PRFe could enhance the cell proliferation and cell migration abilities of SCAPs, with i-PRFe inducing greater cell proliferation than PRFe, and also showed that there was no difference between i-PRFe and PRFe in terms of cell migration. In general, a given cellular response can result from the interaction of multiple cytokines and signaling pathways, ^35^ and the mechanism of action remains to be further investigated.

During the physiological development of tooth roots, stem cells from dental pulp and apical papilla precisely differentiate into odontoblasts by epithelial-mesenchymal interaction, in which situation odontoblast differentiation markers such as *DMP1* , *DSPP* are highly expressed. ^38^ In this study, it was observed that both i-PRFe and PRFe promoted the formation of mineralized nodules and the expression of mineralization-related genes *in vitro* , and i-PRFe gradually obtained an advantage with the increase of induction time. Similarly, previous studies found that the effect of i-PRF was greater than that of conventional PRP in promoting cell mineralization of human dental pulp cells (hDPCs) ^39^ and osteogenic differentiation of osteoblasts. ^20^ The results of this study demonstrated the greater ability of i-PRF to induce odontoblast differentiation of SCAPs, suggesting that i-PRF may also promote the formation of new dentin when applied *in vivo* .

The highly vascularized pulp tissue is located in a confined environment with the small apical foramen being the only means of access to blood supply. Revascularization by reestablishing a biological connection with the surrounding tissue is the basis for successful regeneration of functional vitalized dental pulp. ^40^ Therefore, the ability of i-PRFe and PRFe to promote angiogenesis was compared by tube formation assay and RT-qPCR. Since the growth factors PDGF-BB, TGF-β1 and VEGF detected in i-PRF and PRF all regulate angiogenesis in their own way, AM was not added to the experimental groups. The results of this study showed that i-PRFe induced larger tubular area and upregulated the relative gene expression of *CXCR4* , *VEGF* and *PDGFRA* compared with PRFe. However, among the four groups, the positive control AM group had the best performance, which may be due to the abundant angiogenic growth factors VEGF and b-FGF in AM. These highly expressed angiogenic markers of SCAPs in the i-PRFe group also demonstrated the angiogenic ability of i-PRF. Similar results were shown by Kubesch, et al. ^34^ (2019), who found that reducing the RCF induced greater cell penetration and significantly improved *in vivo* vascularization in an experimental mice model. We believe that further studies related to innervation should be carried out to make cell experiments more complete.

This study revealed that i-PRF was superior to PRF in facilitating cell proliferation, cell mineralization and angiogenesis of SCAPs. To the best of our knowledge, this was the first cell study to compare the regenerative potential of i-PRF and PRF on any cell type. However, the evaluation of i-PRF is deficient in limited cell experiments, and it is still necessary to assess the specific molecular mechanism and effect of clinical transformation of i-PRF on regenerative endodontics for future benefit.

## Conclusion

Despite having limitations, this study revealed that i-PRF has a higher concentration of growth factors and a superior regenerative potential for the behavior of SCAPs compared with PRF, promoting cell proliferation, mineralization and angiogenesis. In addition, this study preliminarily suggests that i-PRF may be a promising scaffold for use in regenerative endodontics.
